# Personalized Drug Dosage – Closing the Loop

**DOI:** 10.1007/s11095-016-2076-0

**Published:** 2016-12-09

**Authors:** Geoffrey T Tucker

**Affiliations:** Department of Human Metabolism, Medical and Biological Sciences, University of Sheffield, Sheffield, UK

**Keywords:** drug dosage, personalized medicine, pharmacogenetics, physiologically-based pharmacokinetic modeling, therapeutic drug monitoring

## Abstract

A brief account is given of various approaches to the individualization of drug dosage, including the use of pharmacodynamic markers, therapeutic monitoring of plasma drug concentrations, genotyping, computer-guided dosage using ‘dashboards’, and automatic closed-loop control of pharmacological action. The potential for linking the real patient to his or her ‘virtual twin’ through the application of physiologically-based pharmacokinetic modeling is also discussed.

## Introduction

The process of drug dosage involves prescription by the physician, dispensing by the pharmacist and administration to or by the patient. Ideally, the outcome in terms of therapeutic effect and safety is evaluated by the physician who might then adjust the dose accordingly. Several iterations may be necessary to finally close this loop in a satisfactory manner. Control engineers would consider this to be a ‘loose loop’ since it contains human beings, whose knowledge, judgement and behavior may not be optimal. Currently, at least in the UK, teaching of the principles of pharmacokinetics and pharmacodynamics to medical students is minimal, although clinical pharmacist are striving to take more responsibility for drug dosage, and patients often forget or otherwise fail to take their medication as directed. In the move towards the introduction of more ‘personalized drug dosage’, the direct measurement of blood drug concentrations as a guide to drug dosage in individual patients (so-called ‘therapeutic drug monitoring’, TDM), point-of-care pharmacogenetic testing and the application of computer-guided dosage using ‘dashboards’ have been advocated as primary approaches while, particularly in anesthesiology and critical care, methods for automatic closed-loop control of pharmacological action have been around for some time. Clearly, the safe and effective management of multidrug dosage of the complex patient with multiple diseases and multiple prescribers will require an integrated view of pharmacology and therapeutics, taking account of demographic, genetic and metabolomic factors as well as disease severity and progression. In this context, linking the real patient to his or her ‘virtual twin’ through physiologically-based pharmacokinetic (PBPK) (and pharmacodynamic) modeling has the potential to predict appropriate individualized drug dosage and to avoid undesired complex drug-drug interactions. However, realizing this dream requires extensive data from each patient if accurate outcomes are to be forecast.

## Adherence

In 2010 it was reported that of the 3.8 billion prescriptions written in the USA every year, over 50% are taken incorrectly or not at all (1), and poor adherence to medication was estimated to cost the healthcare system $290 billion annually (2). Thus, in ambulatory patients incomplete adherence is a primary source of variability in effective drug dosage, suggesting that improvements in medication management might overshadow those made by the application of, for example, genetic knowledge (3). Advances in the development of cheap approaches to the electronic monitoring of drug intake beyond ‘chip-in-a-bottle’ to ‘chip-in-a-pill’ are a possible solution to this problem (4). Also, especially for the treatment of diseases where inadequate dosing can lead to resistance to therapy, such as malaria, the development of innovative oral dosage forms that are retained in the stomach allowing extended drug release into the intestine promise the provision of weekly rather than daily administration (5).

## Exposure *vs* Response

While variability in drug response has both pharmacokinetic and pharmacodynamic elements, the separation of their contributions is not always evident. Clearly, the ultimate response will reflect both exposure and intrinsic activity and direct pharmacodynamic markers have been used for many years, such as glycemia and glycosuria and prothrombin time for monitoring insulin and anticoagulant therapy, respectively. With respect to the role of variable exposure, while clinicians accept that measurement of renal function is often a prelude to dosage adjustment, this does not necessarily extend routinely with regard to other sources of variability in exposure such as those due to drug metabolism and transport. An area in need of further examination is the determination of the extent of intrinsic pharmacodynamic variability (*i.e*., variability in response in different patients at the same level of exposure), allied with enhanced molecular sub-classification of disease and its severity (6).

## Intra-subject variability

The extent of intra-subject variability in drug exposure and response may reflect intrinsic chronological changes in biology as well as extrinsic influences such as feeding and exercise. The former have been studied particularly with respect to cancer chemotherapy, where the clinical relevance of the timing of drug dosage has been established (7). Minor changes in the diurnal level of hepatic CYP3A activity in healthy subjects with standardized intake of food and drink have been described recently using the kinetics of midazolam after intravenous administration to mark enzyme activity, suggesting little clinical significance (8). However, the hepatic extraction ratio of midazolam is about 0.5, such that its clearance after intravenous administration is dependent on liver blood flow as well as intrinsic enzyme activity.

## Therapeutic Drug Monitoring

The principle of using blood drug level measurement as a marker of dosage is based on the recognition of a ‘therapeutic range’ consistent with effective and safe exposure in the population. However, although TDM is implemented for a limited number of compounds with critical, reasonably well-defined ranges of exposure, there are few prospective randomized data to substantiate its cost- effectiveness (9–11). Technological advances, such as lab-on-a-chip analytical methods, providing rapid turnover and accessibility of results may facilitate the more widespread acceptance of TDM and afford a broader vehicle for evaluating its worth (12).

## Pharmacogenetics

Towards the end of the twentieth century many experts asserted that genetic prediction of responsiveness to drugs would reach the medical mainstream within a ten year period. This included an assurance that genotyping for cytochromes P450 would have a significant impact in improving the efficacy of all drug therapy and in reducing the incidence of adverse drug reactions. However, as both Mark Twain and Niels Bohr said “Prediction is very difficult, especially if it is about the future”, and the promise of widespread clinical application of pharmacogenetic testing remains largely unfulfilled. Such tests may be divided into those that aid the selection of a drug and those that aid in selecting its dosage. In general, implementation of the former has been more successful, especially with regard to the choice of anticancer drugs based on tumour genetics. Dose selection of anticancer drugs remains largely determined by body weight or BSA and usually amounts to maximum tolerated dosage, such that pharmacogenetic tests, certainly those predicting exposure, need to have significant added value. As with the application of TDM, evidence of cost – benefit is a primary issue. Even when the results of randomize clinical trials are available, such as those on the impact of CYP2C9 and VKORC1 genotypes on warfarin dosage, conflicting outcomes from either side of the Atlantic do not provide consensus with regard to clinical implementation (13, 14). Nevertheless, continuing pharmacoeconomic assessment is beginning to offer more clarity (15). Any implementation of routine pharmacogenetic testing in selecting the doses of drugs metabolized by CYP2D6 has been even more protracted. Despite the fact that the ‘debrisoquine/sparteine polymorphism’, associated with large differences in exposure and often in response between CYP2D6 ‘poor’ and ‘extensive metabolizers’, was discovered over 40 years ago (16–19) routine genotyping remains confined to only a few specialist centers.

## Computer-Guided Dosing

Early attempts at providing systematic dosage guidance allowing for variability in exposure were based on the development of manual nomograms such as those for gentamicin, which took account of age, weight, sex and serum creatinine (20). Subsequently, pharmacokinetic models have been incorporated into computerized systems as exemplified by the development and routine use (although not in the USA) of target controlled infusion pumps for intravenous anesthetics (21). These devices employ pharmacokinetic – pharmacodynamic link models and BET (‘Bolus-Elimination-Transfer’) functions to approximate square-wave stepping of plasma drug concentration (22–25). However, although demographic information is accommodated, the inclusion of other features that determine variability in exposure is limited. In a wider clinical context dashboard systems are available that improve the utility of TDM information by incorporating adaptive Bayesian approaches to the prediction of exposure, ideally linked to an individual’s clinical and other details in his or her electronic medical record (26, 27). These systems are run through the ‘cloud’ and are capable of including genotype data for speedier optimization of dosage. They do require more than a single trough plasma drug concentration measurement and a validated pharmacokinetic model for each drug. Currently, the clinical use of dashboard systems is limited for all the usual reasons related to cost-benefit, physician acceptance and resource allocation.

## The Virtual Twin

The application of PBPK modeling has come of age in drug development and regulation, reflecting significant advances over the last 15 years in the predictability of overall pharmacokinetic behavior and the extent of drug-drug interactions (DDIs) from physical chemistry and human *in vitro* data and the availability of dedicated software platforms and associated data bases (28, 29). This approach integrates the selection of *stratified* doses based on exposure in patient groups as a function of age, sex, weight, genotypes, race, co-medication, pharmaceutical formulation, obesity, pregnancy and disease. Accomodating these features, with increasing extension to biologics and linkage with pharmacodynamic models, is clearly of benefit in understanding extremes of risk in different patient *populations*. The challenge for the future is to link an *individual* patient to his or her virtual twin within a PBPK modeling framework to provide safe and effective *individualized* dosage as a component of truly personalized drug therapy at the point of care (28, 30). How else, for example, would it be possible to adjust precisely the dosage of drug X metabolized by CYPs 1A2, 2C9 and 3A4 when a potent CYP3A4 inhibitor is added to therapy in a pregnant lady who is a poor metabolizer genotype for CYP2C9 and also has a low constitutive level of CYP1A2? Potentially this could be done on a hand-held device with connection to the ‘cloud’ and knowledge of a ‘therapeutic range’ of blood drug concentrations. As such, therefore, the ‘bottom-up’ PBPK approach integrating diverse sources of prior information is different to actual TDM with adaptive feedback (‘top-down’, based on direct measurement of exposure) since it is predictive rather than retrospective. Combining both ‘bottom-up’ and ‘top-down methods could potentially have synergistic advantages in the rapid optimization of drug dosage, especially since PBPK models are inherently generic and much ‘richer’ in the incorporation of variable patient features than conventional PK models. However, currently there are two major limitations to the practical application of the virtual twin concept, other than the usual ones of evidence of cost-benefit and physician acceptance. The first is that much work still needs to be done on incorporating the impact of patient attributes, particularly of specific diseases, on exposure and response (31). Secondly, for really precise predictions of exposure many disparate pieces of information about the specific patient need to be readily available in order to provide a sufficiently individualized profile. In this context, for example, genotypes for enzymes and transporters will clearly be helpful but even within these bands there remains significant inter-subject variability in exposure and overlap between genotypes (30). With cytochromes P450 how can variability in oxidoreductase protein content and activity be factored in (32, 33), what if there are no known genotypes affecting activity strongly such as in the case of CYP3A4, and what about epigenetic changes (34)? Clearly, it is possible to mark specific enzyme and transporter activity using ‘cocktails’ of probe compounds (35, 36), including CYP3A4 activity with a micro- or even nano-dose of midazolam (37) but, although generically applicable to dosage of a range of drugs, this is invasive and requires appropriate analytical resources with rapid turnaround not available in all centers. Similarly, endogenous markers are used to assess CYP3A4 activity, namely plasma concentrations of 4 beta-hydroxycholesterol and the urinary 6 beta-hydroxycortisol/cortisol ratio (38). However, although they may be useful to mark *changes* in CYP3A4 activity resulting from inhibition or induction, caution should be exercised in using them as markers of absolute (basal) activity since other enzymes /renal clearance influence their values. Indeed 4 beta- hydroxycholesterol is mainly cleared by CYP7A1 which, like 3A4, is regulated by PXR but, unlike 3A4, appears to be downregulated by rifampicin rather than induced by it leading to an increase in its elimination half- life (39–41).

## Automatic Closed-Loop Control

The ultimate in dosage adjustment is an entirely closed loop system based on continuous measurement of a clinically relevant pharmacodynamic end-point with feedback to an administration device. This is exemplified by the automatic control of blood sugar with an implanted glucose sensor and insulin pump (42) and, more recently, using microneedles attached to the skin (43). In anesthesiology and critical care the observation of many drug effects is virtually instantaneous, making the application of control engineering principles with adaptive feedback a particularly attractive possibility in this context. Accordingly, systems for the real time closed-loop administration of intravenous anesthetic agents have and are being evaluated utilizing proportional-integral-derivative (PID controllers that seek a set point based on a processed EEG signal (BIS, bispectral index or auditory evoked response) (44, 45). In this way adaptive controllers are able to self-tune the control parameters during their use as they ‘learn’ more about the individual patient. Variants of these systems also incorporate pharmacokinetic models that select from a predetermined series of target plasma drug concentrations to produce a level of arousal score (46). In post-operative care the use of patient-controlled analgesia *via* an infusion pump is now common (47). This is based on the reasonable premise that the patient, rather than the doctor or the nurse, knows better as to how much morphine he or she needs within the constraint of a set lock-out time. With the increasing development of wireless-based devices that allow continuous monitoring of physiological and biochemical variables with linkage to central data bases, it is likely that these will stimulate further developments in tightening the loop of drug administration as part of a greater alliance between the 3D’s (Diagnostics, Drugs and Devices).

## Epilogue

To the extent that many improvements in drug dosage will depend on solutions that are inherently complex and require special knowledge (e.g., of pharmacokinetics, pharmacodynamics, pharmacogenetics, metabolomics), confidence in their clinical application on the part of the prescriber will clearly depend on appropriate performance verification and a degree of education to allow plausibility checks. While individualized dose predictions can never be exact minimal targets for innovation might be to decrease inter-individual variability in drug exposure to two-fold and ADR’s by half.

## Abbreviations

ADR: Adverse drug reaction
BIS: Bispectral index
BSA: Body surface area
CYP: Cytochrome P450
DDI: Drug – drug interaction
EEG: Electroencephalogram
TDM: Therapeutic drug monitoring
VKORC1: Vitamin K epoxide reductase complex
